# The complete genome sequence of *Vibrio aestuarianus* W‐40 reveals virulence factor genes

**DOI:** 10.1002/mbo3.568

**Published:** 2018-01-03

**Authors:** Xiaojin Xu, Lixing Huang, Yongquan Su, Qingpi Yan

**Affiliations:** ^1^ Fisheries College Key Laboratory of Healthy Mariculture for the East China Sea Ministry of Agriculture Jimei University Xiamen Fujian China; ^2^ State Key Laboratory of Large Yellow Croaker Breeding Ningde Fujian China; ^3^ College of Ocean & Earth Sciences Xiamen University Xiamen Fujian China

**Keywords:** *Penaeus vannamei*, *Vibrio aestuarianus*, virulence, whole‐genome sequence

## Abstract

*Vibrio aestuarianus* is an opportunistic environmental pathogen that has been associated with epidemics in cultured shrimp *Penaeus vannamei*. Hepatopancreas microsporidian (HPM) and monodon slow growth syndrome (MSGS) have been reported in cultured *P. vannamei*. In this study, we sequenced and assembled the whole genome of *V. aestuarianus* strain W‐40, a strain that was originally isolated from the intestines of an infected *P. vannamei*. The genome of *V. aestuarianus* strain W‐40 contains two circular chromosomes of 483,7307 bp with a 46.23% GC content. We identified 4,457 open reading frames (ORFs) that occupy 86.35% of the genome. *Vibrio aestuarianus* strain W‐40 consists primarily of the ATP‐binding cassette (ABC) transporter system and the phosphotransferase system (PTS). CagA is a metabolism system that includes bacterial extracellular solute‐binding protein. Glutathione reductase can purge superoxide radicals ($O_{2}^{2-}$) and hydrogen peroxide (H_2_O_2_) damage in *V. aestuarianus* strain W‐40. The presence of two compete type I restriction‐modification systems was confirmed. A total of 42 insertion sequences (IS) elements and 16 IS elements were identified. Our results revealed a host of virulence factors that likely contribute to the pathogenicity of *V. aestuarianus* strain W‐40, including the virulence factor genes *vacA*,* clpC*, and *bvgA*, which are important for biofilm dispersion. Several bacitracin and tetracycline antibiotic resistance‐encoding genes and type VI secretion systems were also identified in the genome. The complete genome sequence will aid future studies of the pathogenesis of *V. aestuarianus* strain W‐40 and allow for new strategies to control disease to be developed.

## 1 INTRODUCTION

Aquaculture suffers from a number of diseases caused by pathogens (Huang et al., 2016; Kwon, Kim, & Jung, 2016; Luo et al., 2016; Qin, Lin, Chen, Xu, & Yan, 2016). *Vibrio aestuarianus* is a gram‐negative bacterium that is able to survive in diverse marine environment, including oceans, aquaculture farms, and marine invertebrates (Labreuche et al., 2010). Diseases caused by *V. aestuarianus* have posed threats to aquaculture facilities worldwide for 10 years, which has led to the decline of oysters in Japan, the Netherlands, Canada, Spain, France, and the USA (Aboubaker, Sabrie, Huet, & Koken, 2013; Balbi et al., 2013). Outbreaks that resulted in mass mortalities of French juvenile oysters (*Crassostrea gigas*) in the summer were associated with *V. aestuarianus* (Aboubaker et al., 2013).

The virulence mechanisms of *V. aestuarianus* are unknown. *Vibrio aestuarianus* may be an opportunist that cooperates in the killing of hosts. Many questions remain unanswered concerning the virulence factors of *V. aestuarianus,* such as how this bacterium is able to defend itself against the immune systems of marine invertebrates to promote disease processes. *Vibrio aestuarianus* was shown to secrete many virulence factors, such as extracellular products (ECPs) (Labreuche et al., 2010). During infection, *V. aestuarianus* was observed to produce toxins and impaired host functions. In addition, *V. aestuarianus* was shown to be able to degrade the hemocyte oxidative metabolism (Labreuche, Soudant, Goncalves, Lambert, & Nicolas, 2006).

Current sequencing upgrades (PacBio RS II) and comparative functional genomics analyses can help us to identify key factors of bacterial pathogenicity (Cong et al., 2017). Recently, the genomes of a number of clinical and environmental bacterial strains, such as *Vibrio cholera, Vibrio parahaemolyticus*, and *Vibrio alginolyticus*, have had their genomes sequenced (Labreuche et al., 2010; Luo, Yu, Jost, Xu, & Huang, 2015; Yang, Liu, Luo, & Pan, 2015). The zinc metalloprotease of *V. aestuarianus* was shown to promote lethality in oysters (Labreuche et al., 2010; Soudant, Mazel, & Nicolas, 2010). The complete genome sequences of *V. parahaemolyticus*,* V. cholera*, and *V. alginolyticus* have been compared (Kim, Lee, Hee, Nair, & Kim, 2015; Luo et al., 2015; Yang et al., 2015). The genome of another member of the family Vibrionaceae, *V. aestuarianus*, also needs to be sequenced.

In this study, *V. aestuarianus* strain W‐40 was isolated from the intestine of an infected *Penaeus vannamei* specimen and was determined to be a potential conditional pathogen of *P. vannamei*. Diseases caused by *V. aestuarianus* in juveniles and adult oysters have been previously reported (Balbi et al., 2013).

For the first time, we sequenced the complete genome of *V. aestuarianus* strain W‐40 to identify genes related to pathogenicity. Our results revealed a host of virulence factors that likely contribute to the pathogenicity *V. aestuarianus* strain W‐40, including the *vacA, clpC,* and *bvgA* virulence factor genes, type VI secretion systems Mitsutoshi, Jayeeta, & Tamaki, 2014; Huang et al., 2015), and several bacitracin and tetracycline antibiotic resistance‐encoding genes. The major factors in its pathogenic abilities were its intrinsic resistance to antibiotics and other virulence factors. These virulence factors may participate in bacterial pathogenesis through diverse mechanisms. This research will provide an additional tool for in‐depth investigation of the mechanisms involved in the control of diseases.

## 2 MATERIALS AND METHODS

### 2.1 Isolation of bacterial strains

Bacterial strains were isolated from the intestines of infected *P. vannamei*, which were obtained from Xiamen, China. The surfaces of 10 shrimps were disinfected with 70% ethanol. The intestinal tracts of the shrimps were surgically isolated, suspended in PBS and homogenized. After 10‐fold serial dilutions using 1 ml of the suspension, the dilutions were spread on Luria–Bertani agar (LBA) plates + NaCl 0.5 mol/L, after which they were incubated at 28°C for 24 hr. The intestines of healthy *P. vannamei* were added to the growth medium as supplements. The dominant colonies were selected and further isolated by streak plating to obtain pure cultures.

### 2.2 The identification of bacterial strains

The identification of bacteria was conducted using a Biolog Microstation System (Biolog Inc., USA) and 16S rDNA sequence determination. The 16S rDNA sequences from the isolated strains were sequenced using a PCR‐based technique. To amplify partial 16S rDNA fragments from the isolates, universal primers (27F: 5′‐AGAGTTTGATCATGGCTCAG‐3′; 1492R: 5′‐GGATACCTTGTTA CGACTT‐3′) were used. The PCR product was purified and sequenced, and the sequence was aligned at the National Center for Biotechnology Information (NCBI) (http://www.ncbi.nlm.nih.gov). *Vibrio aestuarianus* strain W‐40 stock culture was stored at −80°C.

### 2.3 In vitro challenge of *P. vannamei* with *V. aestuarianus* strain *W‐40*

*Vibrio aestuarianus* strain W‐40 was routinely cultured on LBA plates + NaCl 0.5 mol/L and incubated at 28°C for 24 hr and was stored in our culture collection. Culturing was done using Luria–Bertani (LB) liquid medium with shaking at 28°C for 24 hr. The densities of the bacterial cultures were assessed using a microplate reader (Synergy HT, Bio‐Tek Instruments, Inc., Winooski, VT, USA), with the absorbance measured at 600 nm. *Vibrio aestuarianus* strain W‐40 was used at a final density of 1.35×10^6^ CFU/ml. Adult *P. vannamei* used in the experiment were obtained from Jimei market, Xiamen, China, where the experiment was carried out. *Penaeus vannamei* were examined before the trial to ensure that they were not infected with a pathogen such that (1) there was no abnormal color change in the body in the selected shrimp (e.g., feelers, prawn tails, and appendages) and (2) no dead shrimp were observed and ingestion of the shrimps was normal during the acclimatization period. Prior to experimental challenges, the shrimps were acclimatized for 3 days. *Penaeus vannamei* were randomly placed in each of three aquaria (12 *P. vannamei*s per aquarium) with an flow‐through seawater circuit at 28°C under continuous aeration. In vivo assays were performed on 8‐month‐old *P. vannamei* following previously described procedures (Cameron et al., 2006). For each animal, 200 μl of *V. aestuarianus* strain W‐40 was injected into the muscles of the abdominal segments of *P. vannamei*. Positive and negative control groups were inoculated with 200 μl of untreated PBS, respectively. All experiments were performed in triplicate, and the cumulative mortality was recorded every 4 hr during the experiment.

### 2.4 DNA extraction

The DNA of *V. aestuarianus* strain W‐40 was extracted from pure bacterial colonies for whole genome amplification using the DNA extraction kit D2500‐02 (Omega, Inc., USA) according to the manufacturer's recommendations. The DNA was resuspended in 50 μl of ultra‐pure water and stored at −80°C. DNA yields were detected by spectrophotometry (Eppendorf).

### 2.5 Genome sequencing and assembly

Complete genomic sequencing was performed using PacBio RS II technology, from which 88,518 reads totaling 1,172,325,683 bases (average read length: 13,243 bp, mean read score: 0.84) were obtained, representing a 16‐fold genome coverage. FastqToCA and Celera software packages were used for final assembly and editing, and low‐quality regions of the genome were re‐sequenced. Continuous long reads were obtained from three Single‐Molecule, Real‐Time (SMRT) sequencing runs. Reads longer than 500 bp with a quality value of over 0.80 were merged together into a single dataset. Next, the PBcR pipeline was used to correct for random errors. The longest 25× subset of the corrected data was used for de novo assembly using a Celera Assembler, which employs an overlap‐layout‐consensus (OLC) strategy, with the default parameters.

### 2.6 Genome annotation

Coding sequences were predicted by Glimmer 3.02, and genome annotation was conducted with the basic local alignment search tool (BLAST). An extensive curation of the genes, correction of the start codon positions and the functional assignments was performed. All unigenes were searched in the protein databases using the BLAST ALL procedure with an E‐value <1.0 E^−6^.

The Kyoto Encyclopedia of Genes and Genomes (KEGG), orthologous groups (COG), SwissProt and the NCBI Non‐Redundant Dataset (NR) databases were used to search domain architecture. A BLASTx analysis was used for the SEED subsystems hierarchy at an e‐value cutoff of 1 × 10^−2^. The rRNA gene predictions were performed by RNAmmer 1.2. No gap was then filled in by sequencing. tRNA genes were predicted using the program tRNAscan‐SE 1.21. Finally, virulence and resistance genes were annotated based on the VFDB and ARDB databases. For screening using the VFDB database, virulence genes were annotated and compared according to threshold criteria. A value of e <1‐e^5^ was selected for BLAST searches. Gene islands were predicted using IslandPick, SIGI‐HMM and IslandPath‐DIMOB software in IslandViewer. Insertion sequence (IS) elements were analyzed separately in the IS finder database. Detection and comparative analysis of insert sequences in the IS Finder database used the default parameters and manual selection to identify insertion sequences in the genome.

## 3 RESULTS

### 3.1 Identification of *V. aestuarianus*

In total, many pure cultures were isolated from the intestinal tracts of shrimp. The species of the isolates was determined by 16S rDNA sequence analysis and comparison with sequences in the GenBank databases. The isolates were identified as *V. aestuarianus,* specifically *V. aestuarianus* strain W‐40.

### 3.2 In vivo pathogenicity characterization

**Figure 1:**
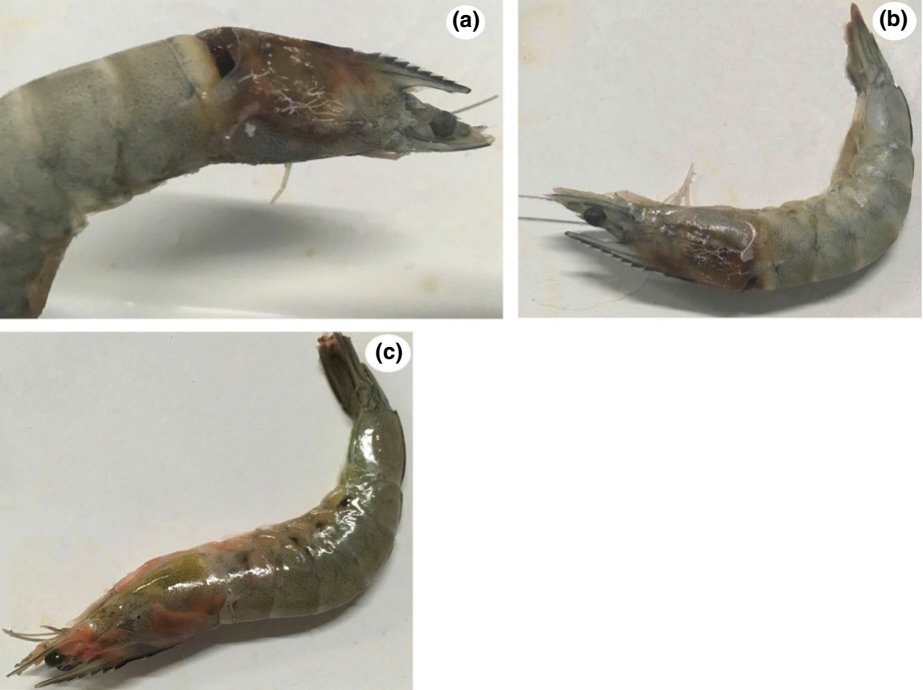
(a, b, c) Typical external signs of *Penaeus vannamei* challenged with *Vibrio aestuarianus*

To assess the virulence of *V. aestuarianus* strain W‐40 to *P. vannamei*, in vivo bacterial injection challenges were performed and were repeated in three independent experiments. As shown in Figure 1, direct muscular injection of the pathogenic strain W‐40 led to 85% *P. vannamei* mortality in approximately 3 days. Cumulative mortality of control *P. vannameis* did not exceed 3% throughout the experiment.

### 3.3 Genome features

**Figure 2:**
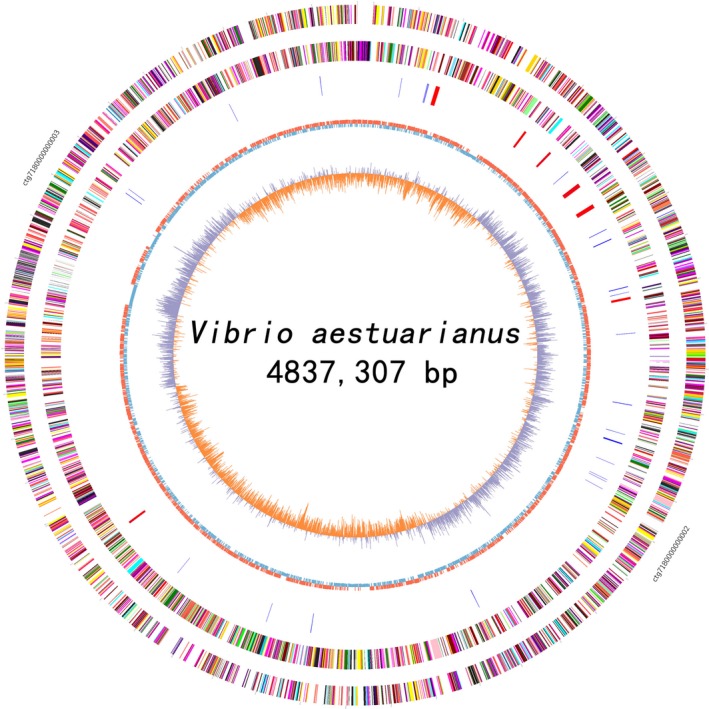
Draft circle genome of *Vibrio aestuarianus* strain W‐40 assembly. The genome consist of chromosome I (ctg7180000000002) and chromosome II (ctg7180000000003). Starting with the outer colored circle, moving inside. The circles depicted coding sequences were distributed in the colored boxes, colored according to different functional categories and direction of transcription. The 1st circle represents the plus strand; the 2nd circle represents the minus strand. The 3rd circle represents tRNAs (blue) and the locations and direction of ribosomal RNA genes (red arrows). The 4th and 5th (innermost) circle indicates mean gene centered G+C content (red plot represents higher than average, the blue plot represents less than average) and GC skew (G − C)/(G + C), sliding window size of 1‐kb, and calculating in steps of 500 bp

The isolate was identified as *V. aestuarianus* strain W‐40 using the Biolog Microstation System and by 16S rDNA sequence determination. *Vibrio aestuarianus* strain W‐40 genome consists of chromosome I (ctg7180000000002), which is 3202,773 bp in length and has a GC content of 46.42%, and chromosome II (ctg7180000000003), which is 1,634,534 bp in length and has a GC content of 45.85%. In total, the genome contains of 4837,307 bp and has a GC content of 46.23% (Figure 2). We identified 4457 ORFs in the genome with an average length of 937 bp. We determined a mean GC content of 46.64% that occupied 86.35% of the genome. Whole genome sequencing generated 1,172,325,683 bases and 88,518 reads and revealed the presence of more than 0.02% adapter dimers (0–10 bp) and 0.01% short inserts (11–100 bp) (Table S1).

**Figure 3:**
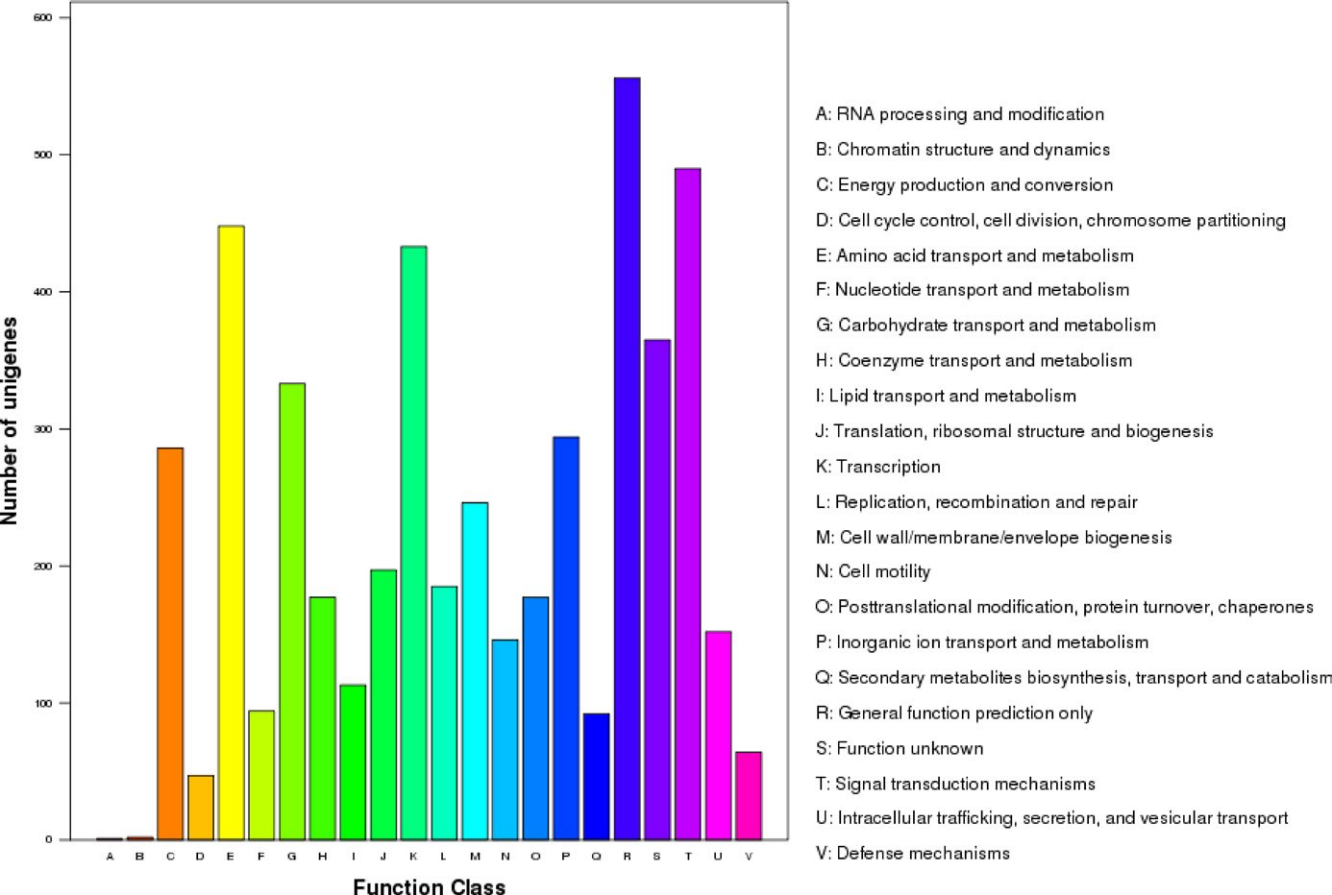
COG Function classification of *Vibrio aestuarianus* strain W‐40. glimmer. cds. fa. Sequcence. A, RNA processing and modification; B, Chromatin structure and dynamics; C, Energy production and conversion; D, Cell cycle control, cell division, chromosome partitioning; E, Amino acid transport and metabolism; F, Nucleotide transport and metabolism; G, Carbohydrate transport and metabolism; H, Coenzyme transport and metabolism; I, Lipid transport and metabolism; J, Translation, ribosomal structure and biogenesis; K, Transcription; L, Replication, recombination and repair; M, Cell wall/membrane/envelope biogenesis; N, Cell motility; O, Posttranslational modification, protein turnover, chaperones; P, Inorganic ion transport and metabolism; Q, Secondary metabolites biosynthesis, transport and catabolism; R, General function prediction only; S, Function unknown; T, Signal transduction mechanisms; U, Intracellular trafficking, secretion, and vesicular transport; V, Defense mechanisms

A total of 4533 genes with biological functions that occupied 92.55% of the genome were identified in *V. aestuarianus* strain W‐40. Among them, 286 genes encoded proteins involved in energy production and conversion, 333 genes encoded carbohydrate transport and metabolism proteins, 433 genes encoded proteins involved in transcription, and 152 genes encoded proteins involved in intracellular trafficking, secretion, and vesicular transport (Figure 2). Among these ORFs, 3528 (72.03%) of genes could be classified into 22 functional categories in COG families (Figure 3).

**Figure 4:**
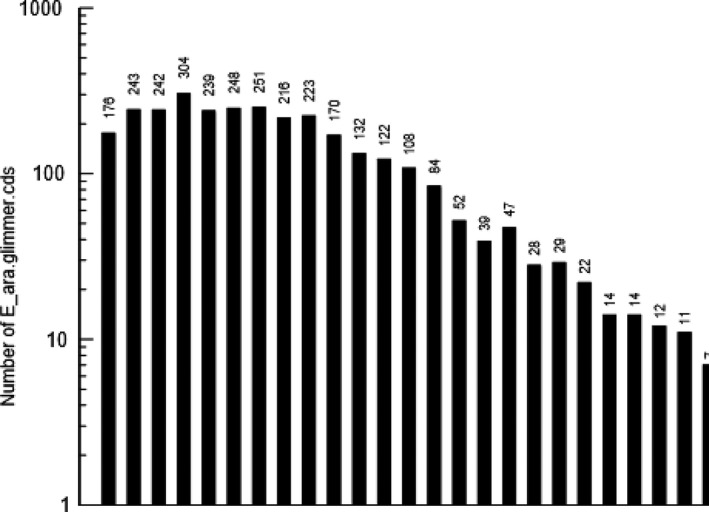
Length distribution of *Vibrio aestuarianus* strain W‐40 glimmer. cds

The length distribution of *V. aestuarianus* strain W‐40 glimmer.cds is shown in Figure 4. The number of *V. aestuarianus* strain W‐40 glimmer.cds obtained as a different sequence size was chosen to group sequences, with the six highest levels of glimmer.cds numbers occurring at sequence sizes of 400–499, 500–599, 600–699, 700–799, 800–899, and 900–999.

In total, 31 rRNA operons were identified, including those encoding 23S, 16S, and 5S rRNAs. The genome encodes 118 tRNAs that represent all 33 amino acids, including tRNA gene types for *met, phe, thr, asn, glu, lys, ala, aal, asp, trp, arg, his, pro, ile, tyr, gly, aer, leu, cys,* and *gln*. tRNA genes included anticodons for *cat, gaa, tgt, gtt, ttc, ttt, tgc, tac, gtc, cca, ccg, gtg, tgg, gat, gta, tcc, ggt, gcc, gct, acg, caa, ggc, gag, tct, gga, gca, taa, gac, ggg, tga, tag, ttg,* and *cct*. In an analysis of the tRNA genes, an intron of the gene was predicted in the tRNAs with anticodons for *phe* (nucleotide positions 160, 414–160, 489) and *glu* (nucleotide positions 671, 144–671, 219) (Table S2).

### 3.4 Genetic and antibiotic resistance analysis

Genetic and functional analysis of the antibiotic resistance of *V. aestuarianus* strain W‐40 revealed *bacA* and *tet34* resistance types. The *bacA* gene was identified (Ko: 0075). The bacitracin resistance genes of *V. aestuarianus* strain W‐40 were identified (Table S3). The tetracycline‐resistant *V. aestuarianus* strain W‐40 was screened for the tetracycline resistance gene *tet34* (Ko: 1124).

### 3.5 Insights into *V. aestuarianus* strain W‐40 Virulence genes

To predict the virulence mechanisms of *V. aestuarianus* strain W‐40, we sought to determine the presence of virulence factors in the genome. Many virulence factors were observed in our study, and the COG, Nr, and KEGG database analyses resulted in the annotation of many virulence factors. The *cagA* genes were confirmed to be present in *V. aestuarianus* strain W‐40 (Ko: 0291, 0506, 0517, 1466, 1606, 3518, and 3811), as were *vacA* genes (Ko: 0282, 0598, 0650, 1119, 3246, 3363, and 3676). The *cagA* and *vacA* genes are coexpressed in most of the strains. These two virulence factors have an effect in the establishment of infection and disease.

The *bvgA* genes (Ko: 1629 and 2622) were identified, with BvgA belonging to a family of regulatory proteins.

The identified virulence factors included hemolysin, and those involved in phospholipase production. Hemolysin was identified in *V. aestuarianus* strain W‐40 (Ko: 1657, 1728, 1788, 2405, 2485, 2904, 2968, 3423, 3778, 3793, 3788, 3991, 4289, 4431, 0060, 0072, 0758, 0889, and 1204). Hemolysin activity is required for leukotriene release, and the virulence factors were primarily annotated as relating to hemolysins, which release inflammatory mediators. The enzyme phospholipase, a secreted virulence factor, was observed in *V. aestuarianus* strain W‐40 (Ko: 1594, 1712, 3803, 3863, 3894, 3894, and 0585). Different virulence factors are regulated differently since, during infection, microorganisms need to produce various virulence factors.

Three lipoproteins in the genome of *V. aestuarianus* strain W‐40 were associated with virulence (Ko: 3301, 3855, W‐4037), and three lipoate protein ligase (LplA)‐encoding genes (Ko: 1733, 3821, and 3820) were annotated. LplA from *V. aestuarianus* strain W‐40 ligates exogenous lipoic acid to the E2 subunit of pyruvate dehydrogenase (PDH) to produce E2‐lipoamide. The *clpC* gene (Ko: 1995, 2995, 3536) was observed in *V. aestuarianus* strain W‐40. The ClpC ATPase helps microorganism to evade host macrophage phagosomes and is necessary for bacterium adhesion and invasion. *Vibrio aestuarianus* strain W‐40 also encodes *clpB* (Ko: 3759, 0508, 0852, 1836, 1841, 1206), which is 50% similar to *clpC*. The gene (Ko: 2684, 3522,0191, 0653, 0679) in *V. aestuarianus* strain W‐40 encodes a trans‐membrane protein that acts on a spermidine/putrescine ABC transporter permease (Table S11). The gene (Ko: 0540, 1905, 2495, 2747) encodes an extracellular protein, while Ko: 4143, 0153, 0532, 1591, and 2401 encode *V. aestuarianus* strain W‐40 cytoplasmic proteins.

A virulence locus in *V. aestuarianus* strain W‐40 encodes a protein secretion apparatus (Ko: 0007, 0099, 1129, 1129, 1816, 2296, 2360, 2360, 3005, 3006, 0158, 0490, 0710, 1004, 1666, 2281, 2386, 2983, 3045, 3122, 3505, 3786, 3938, 4084, and 3720). The production of the fliN‐flagellar motor switch protein was observed in the genome **(**Ko: 0006). FliN was annotated and is known to be required for motility. FliN in the pathogenic *V. aestuarianus* strain W‐40 may have important functions in virulence protein export.

A number of island proteins were annotated, and the cag pathogenicity island protein was identified in the genome (Ko: 0044) and encodes type I‐specific and disease‐associated virulence factors. The *tcpA* gene (Ko: 1542, 2278) encodes the major subunit protein of the toxin coregulated pilus (TCP) in *V. aestuarianus* strain W‐40. The *tcpA* gene was used for the development of the current specific vibrio monitoring system. To investigate the pathogenicity of *V. aestuarianus* strain W‐40*,* we identified many virulence factors among the genes. Some genes with an unknown biological function (i.e., conserved hypothetical proteins) may be related to virulence (Table S4).

*Vibrio aestuarianus* strain W‐40 encodes a number of factors predicted to be important for biofilm production (Ko: 0085, 0155, 1694, 1781, 1830, 1847, 1966) (Table S4).

### 3.6 Metabolism

The genome was observed to contain nine enzymes related to the metabolism of oligopeptides into amino acids to meet the demand for nitrogen for *V. aestuarianus* strain W‐40 (Table S5). Genome analyses using the COG, Nr, and KEGG databases indicated that *V. aestuarianus* strain W‐40 uses glycerol and sugar as carbon sources (Ko: 0020, Ko: 0078). *Vibrio aestuarianus* strain W‐40 assimilates glycerol using two glycerol ABC transport systems and one glycerol uptake facilitator protein (Ko: 2764). Glycerol is altered by glycerol kinase into glycerol 3‐phosphate (Ko: 2765). Glycerol‐3‐phosphate dehydrogenase (Ko: 2766) and triosephosphate isomerase (Ko: 3364) were identified in the genome. Esterase/lipase genes that encode proteins with which bacterial lipase activity were identified in the genome of *V. aestuarianus* strain W‐40. These proteins may provide glycerol for bacterial intracellular life (Ko: 0257). The pyruvate dehydrogenase complex could convert pyruvate into acetyl‐CoA and lactate by D‐lactate dehydrogenase (Ko: 2077, Ko: 4143) and L‐lactate dehydrogenase (Ko: 4149). Acetyl‐CoA is altered into acetyl phosphate by phosphate acetyltransferase (Ko: 1980, Ko: 2096), while acetyl phosphate is transformed into acetate by acetate kinase (Ko: 3630, Ko: 1605), and another ATP molecule is produced (Table S5).

### 3.7 Oxygen stress

Glutathione reductase was identified (Ko: 0600) in W‐40 and was annotated in the COG, Nr, and KEGG databases. This enzyme can purge superoxide radicals ($O_{2}^{2-}$) and hydrogen peroxide (H_2_O_2_) to prevent their associated damage. With respect to dissolved oxygen stress, the primary mechanisms include a thioredoxin (Ko: 1244), a thioredoxin reductase (Ko: 1724), a thiol peroxidase (Ko: 1318) and a peptide methionine sulfoxide reductase (Ko: 2991, Ko: 3835, Ko: 0822) (Table S6). Glycerol 3‐phosphate oxidation may produce H_2_O_2_ that can damage the host cell. However, glycerophosphate oxidase (GlpO) was absent from the *V. aestuarianus* strain W‐40.

### 3.8 The RM system

*Vibrio aestuarianus* strain W‐40 contains two compete type I and VI RM systems (Ko: 0194, 0195, 3063, and 3064) (Table S7). However, the HsdR (Ko: 0193) ortholog of *V. aestuarianus* strain W‐40 is fragmented into pseudogenes (Table S7). A strong barrier for gene transfer is present in *V. aestuarianus* strain W‐40. Gene fragments of the type III RM system methylase gene and the type II RM system endonuclease subunit were absent from the genome.

### 3.9 Evolutionary position

**Figure 5:**
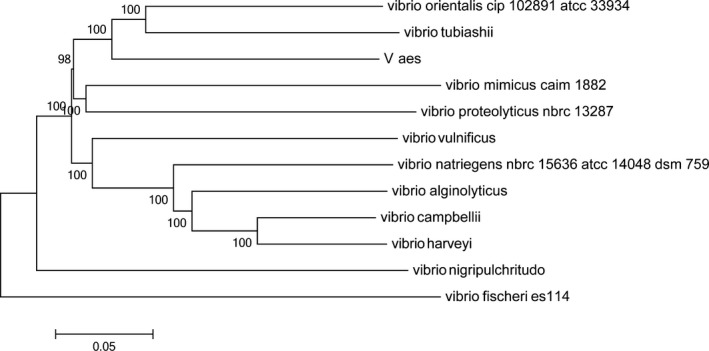
Phylogenetic relationship of strains of *Vibrio aestuarianus* strain W‐40 and related species of other vibrios. Based on complete genome sequences, using MEGA 6. 0 and neighbor‐joining methods. Bootstrap values of support 1,000 times were displayed. The bar indicates 0.05 substitutions per sequence position

We generated a phylogenetic tree using single‐copy orthologous genes that likely well describes the phylogenetic relationship of *Vibrio* species. Twelve orthologous genes from *V. aestuarianus* strain W‐40 and the genomes other *Vibrio* species were used to construct a phylogenetic tree. The position of *V. aestuarianus* strain W‐40 with respect to the phylogenetic relationships of the other *Vibrio* species were generated based on complete genome sequences. The analysis primarily depended on the similarities of single‐copy orthologous genes between the *Vibrio* species. *Vibrio aestuarianus* strain W‐40 was most closely related to *Vibrio tubiashii* and *Vibrio mimicus* caim. We observed single‐copy orthologous genes of *V. aestuarianus* strain W‐40 strains, including the reference strain, with 100% similarities (Figure 5).

### 3.10 IS elements

This is the first report showing complete IS elements in the *V. aestuarianus* strain W‐40 genome. In total, 42 IS elements were identified, such as ISVpa3, ISSoEn2, ISEc39, ISCARN8, and ISWz1. The complete genomic sequencing suggested that ISVpa3, ISVvu5, ISVbsp3, ISVal1, ISVa2, ISVch8, ISV‐M52, ISVba2, ISSpu20, ISSpu11, ISSod12, ISLxx6, IS1237, ISDha14, and ISCARN8 could be grouped to the group IS903 in the IS5 family. IS285, IS1414, ISSod5, ISAs3, ISCARN40, ISSba12, and ISShes5 belong to the IS256 family. ISVpa2 could be classified into the IS3 group within the IS3 family. ISHwa22 and IS200S could be grouped into the IS1341 group in the IS200/IS605 family. The complete IS elements were analyzed. Our findings enrich our knowledge on the insertion sequences of the IS5, IS200, IS256, and Tn3 families (Table S8). These IS elements can serve the basis for future studies concerning interactions of *V. aestuarianus* strain W‐40 and diseases.

### 3.11 IS elements In GIS

A total of 16 IS elements in the GIS genome were identified, including the elements ISSoc10, ISC1332, ISSpn6, ISBj1 ISCARN88. The complete genomic sequencing suggested that ISSoc10, ISHbo2, and ISSpn6 can be grouped into the IS200/IS605 family. ISCsp1 and ISC1332 belongs to the IS256 family. ISBj1 and ISBdi2 could be classified in the IS1380 family, while ISMno32 and ISCARN88 can be grouped into the IS1182 family. ISPfr6 and ISAar42 belong to the ISL3 family. The complete IS elements were analyzed. Our findings enrich our knowledge of the insertion sequences of the IS1380, IS200, IS256,and ISL3 families (Table S9). All of the IS elements in the GIS genome can serve as the basis for future studies concerning interactions of *V. aestuarianus* strain W‐40 and diseases.

### 3.12 Genomic islands analysis

**Figure 6:**
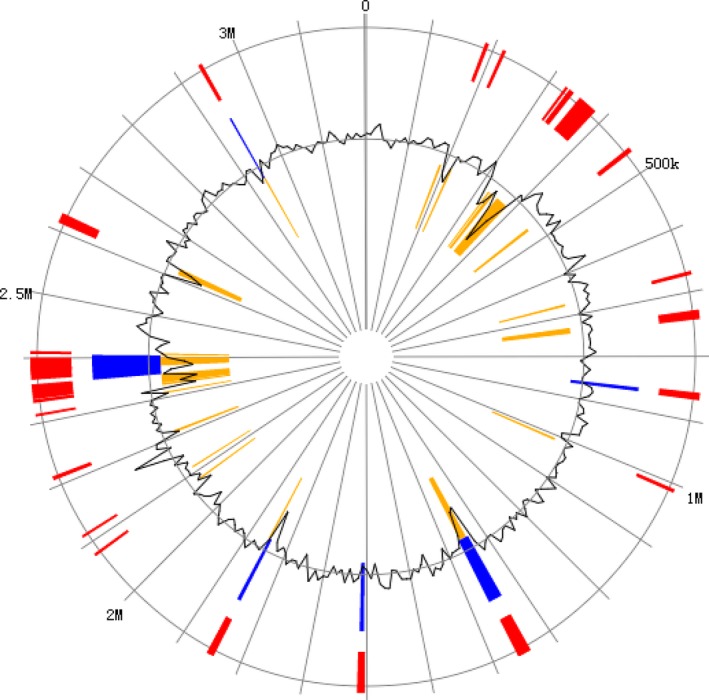
Draft circle GI of *Vibrio aestuarianus* strain W‐40 assembly. Using IslandViewer to predict GI. The circle represents a single chromosome (ctg7180000000002) of *Vibrio aestuarianus* strain W‐40, with red bars around the boundary illustrating all GI predictions using the three methods. In these circles, the predictions of GI are differentiated by prediction method with IslandPath‐DIMOB (blue), SIGI‐HMM (orange), and Islandpick (green) all shown. We can select one result for only one method. There are 18 GIs in the ctg7180000000002 genome of *V. aestuarianus* strain W‐40

**Figure 7:**
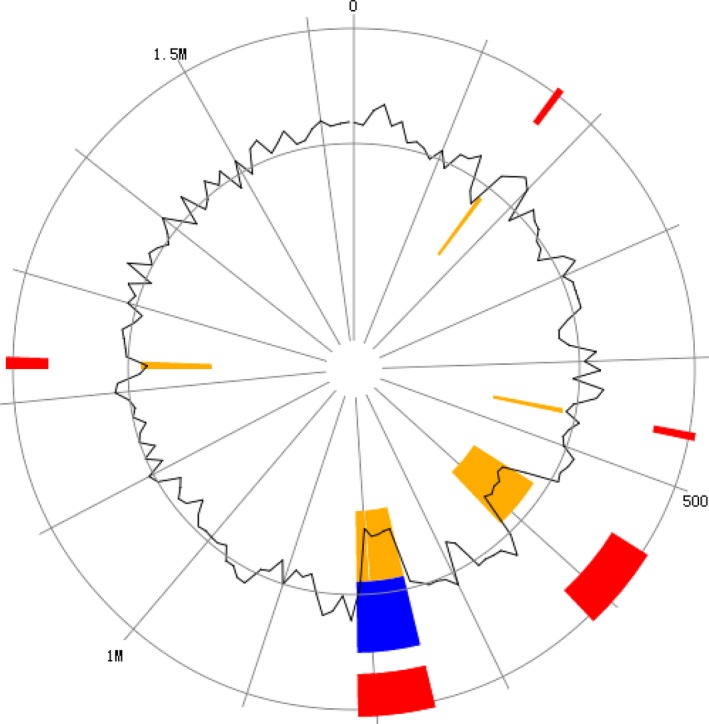
Draft circle GI of *Vibrio aestuarianus* strain W‐40 assembly. Using IslandViewer to predict GI. The circle represents a single chromosome (ctg7180000000003) of *V. aestuarianus* strain W‐40, with red bars around the boundary illustrating all GI predictions using the three methods. In these circles, the predictions of GI are differentiated by prediction method with IslandPath‐DIMOB (blue), SIGI‐HMM (orange), and IslandPick (green) all shown. We can select one result for only one method. There are 5 GIs in the ctg7180000000003 genome of *V. aestuarianus* strain W‐40

The complete genome of *V. aestuarianus* strain W‐40 consists of two single chromosomes (ctg7180000000002 and ctg7180000000003). They are distinguished by a different set of genomic islands: GI0178, GI0179, GI0196, GI0197, GI0198, GI0202, GI0320, GI0199, GI0200, GI0311, GI0312, GI0199, GI0313, GI0341, GI0317, GI0318, GI0319, GI0338, GI0339, GI0340, GI0342, and GI0343 are in a single chromosome (ctg7180000000002) (Figure 6, Table S10), while GI3065, GI3066, GI3067, GI3327, GI3328, GI3329, and GI3330 are in a separate chromosome (ctg7180000000003) (Figure 7, Table S11).

There are 21 GIs in the single chromosome (ctg7180000000002) (Figure 6). GI0178 and GI0179 are capable of directing protein expression to hydrolase (*Haemophilus pittmaniae*). GI0196, GI0197, GI0198, GI0202, GI0311, GI0312, and GI0320 encode hypothetical proteins. GI0341 was identified together with glycosyl transferase function (Table S10).

There are seven GIs in the single chromosome (ctg7180000000003) of *V. aestuarianus* strain W‐40 (Figure 7). GI3066 encodes peptidoglycan‐binding protein LysM. GI3065 is capable of directing protein expression to UDP‐N‐acetylmuramyl peptide synthase (Table S11).

### 3.13 Nucleotide sequence accession numbers

The complete genomic sequence of *V. aestuarianus* strain W‐40 has been deposited in the GenBank database (accession number SRP064227).

## 4 DISCUSSION

To investigate the role of *V*. *aestuarianus* strain W‐40 in *P. vannamei* lethality, we performed toxicity tests. The results showed that, in a short period of time (5 days), the mortality of *P. vannamei* infected with *V*. *aestuarianus* strain W‐40 was up to 85%. Thus, *V*. *aestuarianus* strain W‐40 is weakly pathogenic.

The sequences assembled into two scaffolds and contained 0 gaps, indicating that the entire genome was covered. *Vibrio aestuarianus* strain W‐40 genome consists of chromosome I (3202,773 bp in length) and chromosome II (1634,534 bp in length), which is similar to *V. cholera* (Chaparro, McCulloch, Cerdeira, & Dilaimi, 2011). The similarity in genome size from 30,0000 to 480,000 bp with members of the same genus supports the completeness of our genome sequencing (Kim et al., 2015; Luo et al., 2015; Yang et al., 2015).

It has long been known that toxins and other bacterial virulence factors can allow bacteria to be lethal pathogens. The toxins encoded by *V. aestuarianus* are lethal to hosts. Similarly, toxins of *Vibrio harveyi* were shown to be lethal to the black tiger shrimp (*Penaeus monodon*) (Natrah et al., 2011). *Vibrio aestuarianus* caused massive mortality in the Pacific oyster *C. gigas*, and *V. aestuarianus* has frequently been associated with massive mortality events in *C. gigas* oysters during summer (Labreuche et al., 2010). During many mortality outbreaks, the members of the *Vibrio* genus, including *Vibrio splendidus*, were often detected (Vezzulli et al., 2015; Lacoste et al., 2001; Hua et al., 2015).

The genome of *V. aestuarianus* strain W‐40 contained 100 putative virulence genes that were predicted by a BLASTp search against the Virulence Factors of Bacterial Pathogens database (VFDB), with 143 virulence genes identified in *Piscirickettsia salmonis* (Chen, Xiong, Sun, Yang, & Jin, 2012). *V. aestuarianus* strain W‐40 is similar to *V. cholera*,* V. parahaemolyticus*, and *V. harveyi* with respect to various virulence factors (Kim et al., 2015; Natrah et al., 2011; Yang et al., 2015). The complete genome sequence of *V. aestuarianus* strain W‐40 will provide new insights into the virulence factors and pathogenicity of this bacterium (Goudenège et al., 2015). Extracellular products are known to be related to pathogenesis (Liu et al., 1996; Austin and Zhang, 2006). Moreover, *V. aestuarianus* strain W‐40 lipoate proteins could also be considered to be potential virulence factors. Other identified virulence factors include the *cagA*,* vacA*,* clpC*,* bvgA*, and *lplA* genes loci in *V. aestuarianus* strain W‐40*,* which were expressed in most of the strains (Natrah et al., 2011). In this bacterium, the transcription of virulence‐associated genes is regulated by BvgA proteins, and the BvgAS two‐component system controls the expression of many *bvgA*‐regulated virulence genes (Alice, Qing, & Hinton, 2013). *lplA* plays an important role in aerobic metabolism (Christensen et al., 2013). A virulence locus in *V. aestuarianus* strain W‐40 encodes a protein secretion apparatus, which can help bacterial pathogens to mediate interactions with their hosts (Xu & Liu, 2014). Thus, this apparatus likely contributes to the pathogenesis of *V. aestuarianus* strain W‐40 in the host during chronic infections.

In *V. aestuarianus* strain W‐40, *fliN* (flagellar motor) and extracellular cysteine protease were observed virulence factors. Biofilm formation is very important for CoNS pathogenicity as biofilms allow bacteria to colonize abiotic surfaces, such as indwelling medical devices, which helps to establish infections within the host (Cameron et al., 2015). Similar to *Pseudomonas stutzeri* strains (Pan et al., 2014). *V. aestuarianus* strain W‐40 encodes a type VI secretion system. The type VI secretion system is a highly conserved secretion system used to transport proteins across the bacterial envelope and is precisely regulated in gram‐negative pathogens (Wang, Wen, Li, Zeng, & Wang, 2016). A detailed analysis of this genome containing distinct virulence factors will help in the development of new and efficient vaccines and antimicrobial agents (Beilstein & Dreiseikelmann, 2008; Nonaka & Suzuki, 2002).

The potentials of *bacA* and *tet34* antibiotic resistance genes were identified in *V. aestuarianus* strain W‐40*,* which may suggest that bacitracin‐resistant and tetracycline‐resistant bacteria are aquaculture problems. A major factor in the pathogenicity of a bacterium is its intrinsic resistance to antibiotics and disinfectants. An absence of *bacA* can result in increased bacitracin susceptibility and reduced virulence (Chalker et al., 2000). The over‐expression of *bacA* leads to bacitracin resistance in *Escherichia coli,* since the *bacA* gene encodes a protein that promotes resistance to the antibiotic by phosphorylation of undecaprenol. Bacitracin is clinically used as an antimicrobial drug to inhibit bacterial cell wall synthesis. Similar to *E. coli* and *Bacillus subtilis*,* V. aestuarianus* strain W‐40 can endure higher concentrations of bacitracin (Hachmann et al., 2011). The *tet* gene of *V. aestuarianus* strain W‐40 is responsible for encoding a membrane‐bound undecaprenol kinase responsible for tetracycline resistance. Tetracyclines are broad‐spectrum antibiotics and have been widely used in marine invertebrates for diseases caused by *Acinetobacter baumannii* (Zhu et al., 2014) and *Aeromonas* spp. (Jacob & Chenia, 2007). The determination of intrinsic resistance to antibiotics in *V. aestuarianus* strain W‐40 is important. The study of potential virulence factors could provide insights into the basis of candidates for drug therapy.

*Vibrio aestuarianus* strain W‐40 survival is dependent on the host nutrition for its intracellular lifestyle. Most amino acids, purines, pyrimidines, and cofactors cannot be synthesized *de novo* (Li et al., 2011). This microorganism has high‐affinity transport systems to actively transport solutes across the cytoplasmic membrane, and bacterial extracellular solute‐binding protein was identified in W‐40.

A total of 28 pathogenicity islands were identified in *V. aestuarianus* strain W‐40, which encodes hydrolase, glycosyl transferase, and peptidoglycan‐binding protein. Glycosyl transferase can transform glucose‐1‐phosphate into undecaprenyl pyrophosphate during *E. coli* biosynthesis of the colanic acid exopolysaccharide (Wang et al., 2007). Peptidoglycan‐binding protein Lys is capable of directing protein expression to UDP‐N‐acetylmuramyl peptide synthase (Langille, Hsiao, & Brinkman, 2010). The identification of these genes may lead to a better understanding of the virulence of *V. aestuarianus* strain W‐40.

IS elements are related to lateral gene transfer and genomic evolution. Various IS elements were identified in the IS5, IS256, IS3, IS200/IS605, and Tn3 families in *V. aestuarianus* strain W‐40. IS elements were abundant in the *Mycoplasma bovis* genome (5.4%). IS elements may lead to genomic variation in different strains, which can be horizontally transferred between genomes.

In conclusion, our analysis identified the fliN‐flagellar motor, extracellular cysteine protease, proteins involved in biofilm formation, type VI secretion system and *bacA* and *tet34* antibiotic resistance genes, along with other several predicted virulence factor‐encoding genes in *V. aestuarianus* strain W‐40. The complete sequence results provide new insights into the biology and prevalence of this strain, and new strategies to control vibriosis caused by *V. aestuarianus* strain W‐40, thereby reducing economic losses.

## CONFLICT OF INTEREST

The authors declare that the research was conducted in the absence of any commercial or financial relationships that could be construed as a potential conflict of interest.
